# Retention Intention of Clinical Nurses Returning from Parental Leave in South Korea: A Structural Equation Modeling Study

**DOI:** 10.3390/healthcare14193219

**Published:** 2026-09-30

**Authors:** Jihyun Kim, Mihae Im

**Affiliations:** 1 Department of Nursing, Gwangju University, Gwangju 61743, Republic of Korea; kjh@gwangju.ac.kr; 2 Department of Nursing, Daegu Haany University, Gyeongsan-si 38610, Republic of Korea

**Keywords:** parental leave, nurses, retention intention, work–family conflict, resilience

## Abstract

**Highlights:**

**What are the main findings?**

- Positive affect and job satisfaction were directly associated with retention intention among nurses returning from parental leave.
- Resilience and work–family balance conflict showed significant indirect and total associations with retention intention, and the respecified model explained 52% of the variance in retention intention.

**What are the implications of the main findings?**

- Supporting nurses’ psychological resources and reducing work–family balance conflict may facilitate successful reintegration and long-term retention after parental leave.
- Nursing managers should provide structured return-to-work support, flexible scheduling, and family-friendly workplace policies to promote positive work experiences and retention.

**Abstract:**

**Purpose**: This study aimed to test a hypothesized structural model explaining retention intention among nurses returning from parental leave based on the Job Demands–Resources framework. **Methods**: A cross-sectional survey was conducted between January and April 2019 with 196 nurses who had returned to work after parental leave from 13 general hospitals in Korea. Data were analyzed using SPSS 22.0 and AMOS 21.0. The hypothesized model included parenting stress, spousal support, and work–family balance conflict as exogenous variables; resilience, positive affect, and job satisfaction as mediating variables; and retention intention as the outcome variable. Structural equation modeling was used to assess model fit and test the hypothesized pathways. **Results**: The respecified model showed improved but marginally acceptable fit compared with the initial hypothesized model (χ^2^/df = 2.004, SRMR = 0.067, RMSEA = 0.072, GFI = 0.798, NFI = 0.821, TLI = 0.887, CFI = 0.900). Positive affect and job satisfaction showed significant positive direct associations with retention intention. Resilience and work–family balance conflict showed significant indirect and total associations with retention intention, despite nonsignificant direct associations. Spousal support showed a significant indirect, but not direct, association with retention intention. The respecified model accounted for 52% of the variance in retention intention. **Conclusions**: Retention intention among nurses returning from parental leave was directly associated with positive affect and job satisfaction, whereas resilience, work–family balance conflict, and spousal support showed important indirect associations. Nursing managers may consider strategies to reduce work–family balance conflict and support positive psychological resources and reintegration, including structured return-to-work programs, mentoring, flexible scheduling, equitable workload distribution, and family-friendly workplace policies.

## 1. Introduction

The global healthcare system is facing a critical workforce shortage, with the World Health Organization projecting a deficit of approximately 18 million healthcare workers by 2030 [1]. Within this broader workforce crisis, the nursing profession is particularly affected, with the International Council of Nurses estimating a global shortage of up to 13 million nurses by 2030 [2]. In Korea, the shortage of hospital nurses remains pronounced, characterized by insufficient staffing and persistently high turnover rates compared with other countries [3]. Recent data show that 26.4% of newly hired nurses in Korea leave within their first year [3], highlighting ongoing workforce instability. Moreover, Korea recorded the lowest total fertility rate globally in 2023 (0.72 births per woman), raising concerns about the future supply of healthcare professionals, including nurses [4]. Given these demographic and workforce challenges, retaining experienced clinical nurses has become a critical concern for maintaining healthcare system stability and sustainability.

Approximately 95% of hospital nurses in Korea are female [3], and parental leave following childbirth is predominantly taken by women. Returning to work after parental leave therefore represents a key workforce transition, in which experienced nurses re-enter clinical practice [5]. Unlike newly graduated nurses, returning nurses possess accumulated clinical knowledge, practical competencies, and organizational experience, making them valuable assets to healthcare institutions [5,6]. Retaining these nurses is essential, as their departure results not only in workforce shortages but also in loss of expertise and increased recruitment and training costs [7]. Therefore, successful reintegration and retention of nurses returning from parental leave should be considered a strategic priority for sustaining workforce stability and quality.

Nurses returning from parental leave often face substantial challenges during their transition back to practice. After a period of absence, they must readapt to rapidly evolving clinical environments, new technologies, increased administrative demands, and modified workplace routines [6,8]. In shift-based settings with inflexible schedules and heavy workloads, these challenges may be intensified. Simultaneously, returning nurses must manage parenting responsibilities alongside professional roles, leading to increased parenting stress and work–family balance conflict [8,9]. Previous studies consistently identify work–family balance conflict as a major factor reducing retention intention among nurses returning to work [8,9], while parenting burden is also linked to workforce reintegration difficulties [5]. In Korea, where women often bear substantial caregiving responsibilities, returning nurses experience compounded pressures related to childcare, household roles, and professional adaptation [8].

Despite prior research identifying factors associated with retention among nurses returning to work, several limitations remain. First, most studies focus on individual variables, such as work–family balance conflict or parenting burden, without adequately considering the complex interplay among occupational, familial, and psychological factors [9,10]. Second, although work–family balance conflict is a well-established major risk factor for reduced retention intention, less attention has been given to protective resources that may support reintegration, such as spousal support and individual resilience [8,10]. Spousal support may reduce caregiving burden and perceived work–family balance conflict [11], whereas resilience may enhance adaptive coping during the return to practice. However, these factors are rarely examined together within an integrated framework. Third, most prior studies rely on correlational designs, thereby limiting understanding of the structural associations between job demands, personal and external resources, and retention intention among nurses returning to work [9,10]. Finally, structural model-based studies specifically targeting returning nurses remain scarce.

Retention intention among nurses returning to work may be associated with dynamic interactions between occupational demands and available personal and social resources. The reintegration process involves not only workplace demands but also parenting stressors and family responsibilities, requiring a comprehensive approach that integrates job, family, and psychological factors. Examining the structural relationships among these factors may provide a more comprehensive understanding of retention intention. Therefore, this study aimed to test a hypothesized structural model explaining retention intention among clinical nurses returning from parental leave in South Korea by integrating parenting stress, work–family balance conflict, spousal support, resilience, positive affect, and job satisfaction within a unified explanatory framework.

## 2. Materials and Methods

### 2.1. Study Design

This cross-sectional study employed structural equation modeling (SEM) to test a hypothesized structural model explaining retention intention among clinical nurses returning from parental leave and was reported in accordance with the Strengthening the Reporting of Observational Studies in Epidemiology (STROBE) guidelines [12].

### 2.2. Theoretical Framework and Hypothesized Model

This study was grounded in the Job Demands–Resources (JD–R) model proposed by Bakker and Demerouti [13,14], which explains employee well-being and organizational outcomes as the result of interactions between demands and resources in the work environment. According to the JD–R model, excessive job demands deplete physical and psychological energy, leading to strain and negative occupational outcomes, whereas job and personal resources promote adaptation, motivation, and retention [13,14].

Among nurses returning from parental leave, parenting stress was conceptualized as a family-related demand, whereas work–family balance conflict was conceptualized as a work–family interface strain arising from incompatibility between work and family roles. Spousal support was conceptualized as a family-related social resource that may reduce caregiving burden and facilitate adaptation during the return-to-work transition. Resilience and positive affect were conceptualized as personal resources that facilitate successful adaptation to workplace transitions. Job satisfaction was included as a proximal attitudinal factor reflecting overall positive evaluations of job and organizational experiences.

Based on the JD–R framework, a hypothesized structural model was developed to examine how demands and resources across the work and family domains are associated with retention intention among nurses returning from parental leave. In this model, parenting stress, spousal support, and work–family balance conflict were specified as exogenous variables, whereas resilience, positive affect, job satisfaction, and retention intention were specified as endogenous variables. Direct paths to retention intention were specified from parenting stress, spousal support, work–family balance conflict, resilience, positive affect, and job satisfaction, while resilience, positive affect, and job satisfaction were also positioned as intermediate variables through which demands and resources may be associated with retention intention. The proposed structural relationships are presented as the following hypotheses:

**H1.**  *Parenting stress is negatively associated with resilience.*

**H2.**  *Spousal support is positively associated with resilience.*

**H3.**  *Work–family balance conflict is negatively associated with positive affect.*

**H4.**  *Resilience is positively associated with positive affect.*

**H5.**  *Work–family balance conflict is negatively associated with job satisfaction.*

**H6.**  *Resilience is positively associated with job satisfaction.*

**H7.**  *Positive affect is positively associated with job satisfaction.*

**H8.**  *Parenting stress is negatively associated with retention intention.*

**H9.**  *Spousal support is positively associated with retention intention.*

**H10.**  *Work–family balance conflict is negatively associated with retention intention.*

**H11.**  *Resilience is positively associated with retention intention.*

**H12.**  *Positive affect is positively associated with retention intention.*

**H13.**  *Job satisfaction is positively associated with retention intention.*

### 2.3. Setting and Sample

Participants were recruited through convenience sampling from 13 general hospitals with more than 300 beds in Seoul, Ulsan, Busan, and Gyeongnam in Korea. Eligible participants were clinical nurses who had returned to work for at least 3 months after parental leave taken in accordance with legal criteria for employees with children aged 8 years or younger or enrolled in the second grade of elementary school or below. The 3-month criterion was based on prior research indicating that organizational adaptation and attitudes are generally established after 3 months of employment [15]. Nurses whose children had received treatment for chronic illness or physical or mental disabilities for >3 months were excluded based on previous evidence of differences in parenting stress between mothers of children with disabilities and mothers of children without disabilities [16]. This criterion was applied to reduce heterogeneity in parenting stress associated with additional caregiving demands. The target sample size was determined based on established recommendations for covariance-based structural equation modeling. Hoelter [17] suggested that approximately 200 cases constitute an appropriate sample size for SEM, and Woo [18] similarly recommended a sample of approximately ≥200 when maximum likelihood estimation is used. Accordingly, a minimum sample of 200 participants was targeted. Allowing for an anticipated 10% rate of incomplete or ineligible responses, 220 questionnaires were distributed. All 220 questionnaires were returned. Of these, 24 questionnaires were excluded: 5 because of survey discontinuation, 17 because of missing responses, and 2 because of failure to meet the eligibility criteria, resulting in a final analytic sample of 196 participants. As an additional model-specific evaluation, statistical power was assessed in R using an RMSEA-based approach. Assuming a close-fit RMSEA of 0.05, an alternative RMSEA of 0.08, α = 0.05, 333 degrees of freedom, and N = 196, the estimated statistical power exceeded 0.99, supporting the adequacy of the final sample for evaluating overall model fit.

### 2.4. Measurements

#### 2.4.1. General Characteristics

Participant general characteristics included seven demographic, work-related, and parental leave-related variables: age, educational level, position, clinical experience, number of children, duration of parental leave, and time since returning to work.

#### 2.4.2. Spousal Support

Spousal support was measured using the Korean version of the Parenting Alliance Inventory, validated by Kim et al. [19] based on the original instrument by Abidin and Brunner [20] and supplemented with items from Shin [21]. The instrument includes 14 items across two subdomains: participation in childcare (7 items) and agreement with spouse on parenting (7 items). Items are rated on a 5-point Likert scale, with higher scores indicating greater perceived spousal support.

The reliability of the original instrument was given by Cronbach’s α of 0.93 [20] and 0.92 in the study by Kim et al. [19]. In this study, Cronbach’s α for the total scale was 0.93; subdomain values were 0.92 (participation in childcare) and 0.88 (agreement with spouse on parenting).

#### 2.4.3. Parenting Stress

Parenting stress was measured using the scale developed and validated in Korean by Kim and Kang [22]. The instrument consists of 32 items across three subdomains: daily stress related to parenting (12 items), burden and distress related to the parental role (12 items), and guilt related to childcare provided by others (8 items). Items are rated on a 5-point Likert scale, with higher scores indicating greater parenting stress.

Based on item reliability and confirmatory factor analysis (CFA), two items (Items 21 and 32) with factor loadings < 0.40 were excluded. The original reliability was given by Cronbach’s α = 0.93 [22]. After item deletion, Cronbach’s α for the total scale was 0.95; subdomain values were 0.90 (daily stress), 0.88 (burden and distress), and 0.88 (guilt).

#### 2.4.4. Work–Family Balance Conflict

Work–family balance conflict was measured using the scale developed by Netemeyer et al. [23] and validated in Korean by Kwon and Choi [24]. The instrument includes 10 items across two subdomains: work-to-family conflict (5 items) and family-to-work conflict (5 items). Items are rated on a 5-point Likert scale, with higher scores indicating greater conflict between work and family roles in both directions. In the SEM, work–family balance conflict was specified as a latent construct using the work-to-family conflict and family-to-work conflict subscale scores as two observed indicators; therefore, it was not modeled as a higher-order latent construct.

Original reliability was given by Cronbach’s α = 0.86 [23]. In the study by Kwon and Choi [24], Cronbach’s α values were 0.90 (work-to-family conflict) and 0.86 (family-to-work conflict). In the present study, Cronbach’s α for the total scale was 0.87; subdomain values were 0.82 and 0.81 for work-to-family and family-to-work conflicts, respectively.

#### 2.4.5. Resilience

Resilience was measured using the instrument developed by Park and Park [25] for nurses. It includes 30 items across five subdomains: dispositional (5 items), relational (4 items), situational (10 items), philosophical (6 items), and professional (5 items) patterns. Items are rated on a 5-point Likert scale, with higher scores indicating greater resilience.

The original reliability was given by Cronbach’s α = 0.95 [25]. In the present study, Cronbach’s α for the total scale was 0.97; subdomain values were 0.83 (dispositional), 0.81 (relational), 0.91 (situational), 0.89 (philosophical), and 0.81 (professional).

#### 2.4.6. Positive Affect

Positive affect was measured using the Korean version of the Positive and Negative Affect Schedule (PANAS), originally developed by Watson et al. [26] and validated by Lee et al. [27]. The original instrument consists of 20 items comprising two subscales: positive affect (10 items) and negative affect (10 items). In the present study, only the positive affect subscale was used. Consistent with the Korean validation study, the item “alert” was excluded because it was classified as a negative affect item in the Korean context and demonstrated a relatively low factor loading (0.305), suggesting difficulty in conveying the intended meaning as a positive affect item [27]. Therefore, positive affect was assessed using the remaining nine items. Items were rated on a 5-point Likert scale, with higher scores indicating greater positive affect. The original reliability reported by Lee et al. [27] was given by Cronbach’s α = 0.86 for the positive affect subscale and 0.84 for the negative affect subscale. In the present study, Cronbach’s α for the revised nine-item positive affect scale was 0.89. Positive affect was specified as a latent variable measured by nine observed indicators in the SEM.

#### 2.4.7. Job Satisfaction

Job satisfaction was measured using a single-item overall job satisfaction measure from Scarpello and Campbell [28]. The item asked, “How satisfied are you with your job in general?” and was rated on a 5-point scale ranging from 1 (very dissatisfied) to 5 (very satisfied), with higher scores indicating greater job satisfaction. Subsequent research has provided psychometric support for the use of single-item measures of overall job satisfaction [29]. A single global item was used because the present study focused on overall job satisfaction rather than satisfaction with specific job facets. Job satisfaction was specified as an observed variable in the SEM.

#### 2.4.8. Retention Intention

Retention intention was measured using the Nurses Retention Index developed by Cowin [30] and validated in Korean by Kim [31]. The instrument includes six items rated on an 8-point Likert scale. The two negatively worded items (Items 3 and 6) were reverse-coded before analysis so that higher scores consistently indicated greater retention intention. Although the items included both organizational and professional aspects of retention, they were analyzed as a single construct consistent with the original validation of the Nurses Retention Index. In the present CFA, all six items were loaded on a single latent factor, supporting their treatment as a single retention intention construct.

Original reliability was indicated by Cronbach’s α of 0.97 and 0.88 in the study by Kim [31]. In the present study, Cronbach’s α was 0.91.

### 2.5. Data Collection and Ethical Consideration

This study followed the ethical principles of the Declaration of Helsinki and was approved by the Institutional Review Board (IRB No. HYI-18-195-3) of Hanyang University. Data were collected from 26 January to 10 April 2019.

After obtaining institutional permission, the study purpose and procedures were explained to nursing education and unit managers, and approval was obtained. Participation was voluntary, and participants were informed of their right to withdraw at any time. Anonymity and confidentiality were ensured.

Written informed consent was obtained directly from participants or via questionnaire distribution by head nurses. Participants completed the questionnaires independently and returned them in sealed envelopes. Questionnaire completion required approximately 30 min. Participants received a small gift voucher (approximately KRW 5000) as compensation.

### 2.6. Data Analysis

Data were analyzed using SPSS version 22.0 and AMOS version 21.0 (IBM Corp., Armonk, NY USA). Descriptive statistics (frequencies, means, and standard deviations) were used to summarize participant characteristics and variables. Reliability was evaluated using Cronbach’s α, and validity of latent variables was assessed through CFA. Indicators with standardized factor loadings < 0.40 were excluded during measurement model evaluation. Indicators with loadings above this criterion were retained when they remained theoretically relevant to the construct, with particular consideration given to preserving the content validity of the original validated instruments. Pearson’s correlation coefficients were used to examine relationships among variables, and multicollinearity was assessed using correlation coefficients, tolerance, and variance inflation factors (VIFs).

SEM was performed to test the hypothesized model using maximum likelihood estimation. Prior to model estimation, univariate normality was assessed using skewness and kurtosis, and multivariate normality was assessed using Mardia’s multivariate kurtosis. The multivariate kurtosis coefficient was 69.826 (critical ratio = 11.925), indicating deviation from multivariate normality. Therefore, bias-corrected bootstrap procedures with 5000 resamples were applied to obtain more robust confidence intervals and *p* values for the direct, indirect, and total effects in the presence of non-normality. Following estimation of the hypothesized model, modification indices were examined to identify potential sources of localized model misfit. Model re-specification was conducted post hoc based on model diagnostics and a substantive evaluation of item content. Residual covariances were permitted only when they were supported by substantive overlap in item content rather than on statistical criteria alone. No structural paths were added or removed on the basis of modification indices. Model fit was evaluated using χ^2^, χ^2^/df, root mean square residual (RMR), goodness-of-fit index (GFI), root mean square error of approximation (RMSEA), normed fit index (NFI), comparative fit index (CFI), and Tucker–Lewis index (TLI). A *p* value < 0.05 was considered statistically significant. For indirect effects, statistical significance was also assessed using the 95% bias-corrected bootstrap confidence intervals; an indirect effect was considered statistically significant when the corresponding confidence interval excluded zero.

## 3. Results

### 3.1. General Characteristics of Participants

The mean age of participants was 35.56 ± 4.57 years, with the largest age group being 36–39 years (37.2%), followed by 31–35 years (36.7%). More than half of the participants held a university degree (53.6%). Most participants were staff nurses (64.3%), and the mean clinical experience was 11.68 ± 4.45 years, with the largest proportion having 10–14 years of experience (43.4%).

More than half of the participants had one child (59.2%). Moreover, 41.3% had taken parental leave for ≥12 months, followed by 28.6% who had taken 7–11 months of leave. The mean duration since returning to work was 1.77 ± 0.76 years, and 42.9% had returned within the past year (Table 1).

### 3.2. Descriptive Statistics and Normality of Variables

Among the variables, retention intention showed the highest mean score (5.43 ± 1.39), followed by spousal support (3.66 ± 0.69), resilience (3.64 ± 0.50), work–family balance conflict (3.34 ± 0.62), job satisfaction (3.16 ± 0.71), parenting stress (3.21 ± 0.67), and positive affect (2.43 ± 0.73). Skewness values ranged from −1.01 to 0.39, and kurtosis values ranged from −1.17 to 1.42, indicating that the univariate normality assumption was met (Table 2).

### 3.3. CFA and Multicollinearity of Variables

To evaluate construct validity, a measurement model was specified using multiple indicators for parenting stress, spousal support, work–family balance conflict, resilience, positive affect, and retention intention. Job satisfaction was specified as an observed variable because it was measured using a single item.

CFA showed that AVE values ranged from 0.48 to 0.86. Although the AVE for positive affect (0.48) was slightly below the recommended criterion of 0.50, its construct reliability was 0.89 (>0.70), providing partial support for convergent validity; therefore, the evidence for convergent validity should be interpreted with caution [32]. Overall, construct reliability values ranged from 0.77 to 0.97, supporting the convergent validity of the measurement model. For discriminant validity, squared correlations among the study constructs ranged from 0.04 to 0.32, and all were lower than the corresponding AVE values (0.48–0.86), indicating adequate discriminant validity (Table 2). In addition, HTMT values were all <0.85, further supporting discriminant validity.

Correlations among variables did not exceed 0.85. Tolerance values ranged from 0.26 to 0.86, and variance inflation factors ranged from 1.16 to 3.87, indicating no evidence of multicollinearity.

### 3.4. Model Fit and Model Respecification

The initial hypothesized structural model showed a less-than-optimal fit to the data: χ^2^ = 714.327 (*p* < 0.001), χ^2^/df = 2.132, RMR = 0.075, SRMR = 0.069, RMSEA = 0.076, GFI = 0.779, NFI = 0.809, TLI = 0.872, and CFI = 0.887. Given that several fit indices were below commonly recommended levels, modification indices were examined together with the substantive content of the corresponding items.

Two residual covariances were subsequently permitted between items within the same constructs as post hoc model modifications. For positive affect, the residuals of the items “excited” and “strong” were allowed to covary based on the modification index (MI = 25.791) and their similar content reflecting high-activation positive affect (standardized residual correlation = 0.385, *p* < 0.001). For retention intention, the residuals of Items 3 and 6 were allowed to covary based on the MI (18.509) and because both items reflect intentions to leave the nursing profession (standardized residual correlation = 0.328, *p* < 0.001).

Following these modifications, all examined fit indices improved: χ^2^ = 667.252 (*p* < 0.001), χ^2^/df = 2.004, RMR = 0.070, SRMR = 0.067, RMSEA = 0.072, GFI = 0.798, NFI = 0.821, TLI = 0.887, and CFI = 0.900 (Table 3). Although the respecified model showed improved fit relative to the hypothesized model, several incremental fit indices, including GFI, NFI, and TLI, remained below conventionally recommended thresholds, and the CFI of 0.900 should be interpreted as borderline rather than indicative of strong model fit. Overall, the respecified model was therefore considered to have improved but marginally acceptable fit.

### 3.5. Structural Effects of the Respecified Model

**Figure 1:**
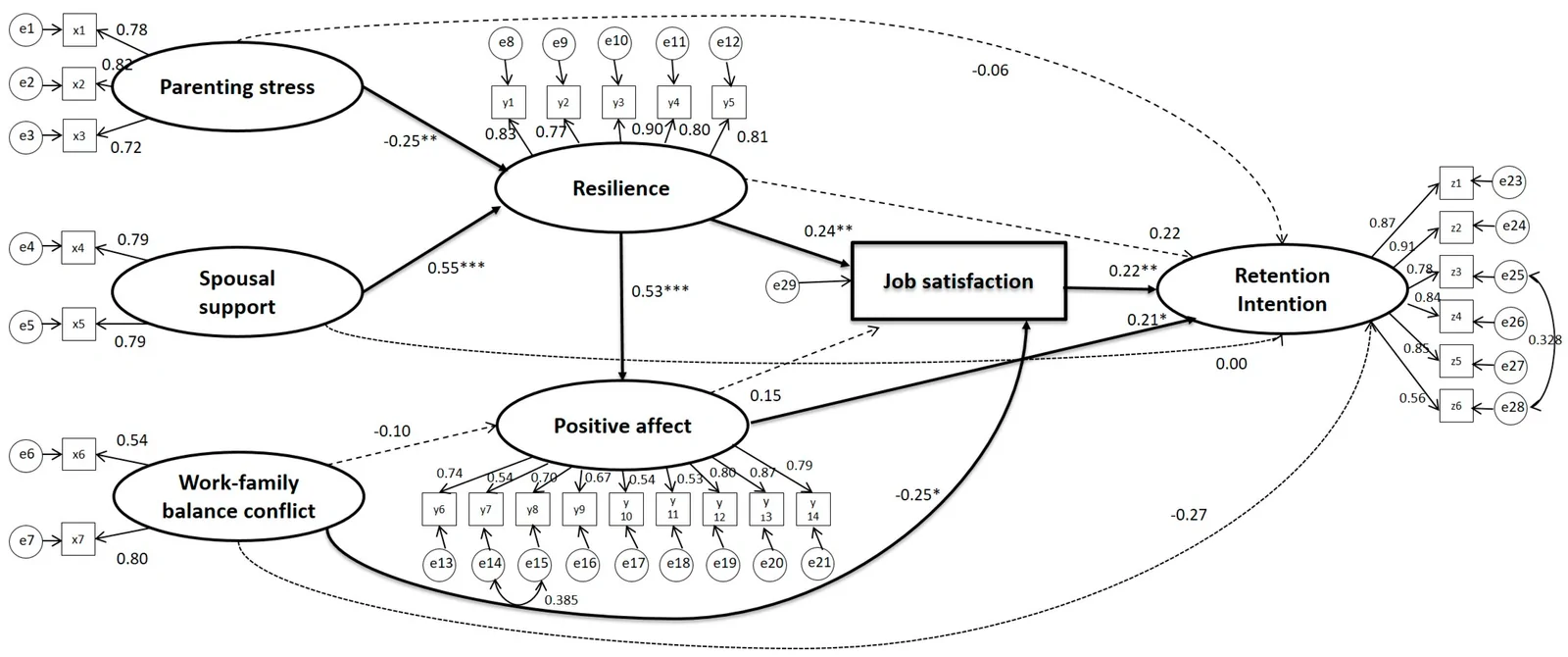
Respecified structural model. Note. Ellipses represent latent constructs, and rectangles represent observed variables. Job satisfaction was measured using a single item and was therefore modeled as an observed variable. Values on measurement paths represent standardized factor loadings, and values on structural paths represent standardized path coefficients. Solid lines indicate statistically significant structural paths, whereas dashed lines indicate nonsignificant paths. Curved double-headed arrows indicate correlated residuals. * *p* < 0.05, ** *p* < 0.01, *** *p* < 0.001. x1: Daily stress related to parenting; x2: Burden and distress related to the parental role; x3: Guilt related to childcare provided by others; x4: Participation in childcare; x5: Agreement with spouse on parenting; x6: Work-to-family conflict; x7: Family-to-work conflict; y1: Dispositional pattern; y2: Relational pattern; y3: Situational pattern; y4: Philosophical pattern; y5: Professional pattern; y6: Interested; y7: Excited; y8: Strong; y9: Enthusiastic; y10: Determined; y11: Attentive; y12: Inspired; y13: Active; y14: Proud. z1: I intend to continue working at my current hospital. z2: I plan to remain in my current hospital as long as possible. z3: I plan to leave nursing in the near future. z4: I want to continue working as a nurse. z5: I will continue working at my current hospital whenever possible. z6: I would like to find a profession other than nursing.

In the respecified model, 7 of the 13 hypothesized structural paths were statistically significant (Figure 1). Regarding the total associations with retention intention, resilience showed the largest positive total association (β = 0.40, *p* = 0.001), whereas work–family balance conflict showed a significant negative total association (β = −0.34, *p* = 0.033). Positive affect (β = 0.24, *p* = 0.007) and job satisfaction (β = 0.22, *p* = 0.007) also showed significant positive total associations with retention intention.

Work–family balance conflict showed a significant negative indirect association with retention intention (β = −0.08, *p* = 0.031) and a significant negative total association (β = −0.34, *p* = 0.033), whereas its direct association was not statistically significant (β = −0.27, *p* = 0.091). Resilience showed a significant positive indirect association (β = 0.18, *p* < 0.001) and a significant positive total association (β = 0.40, *p* = 0.001), although its direct association with retention intention was not statistically significant (β = 0.22, *p* = 0.087). Positive affect demonstrated a significant positive direct association (β = 0.21, *p* = 0.014) and a significant positive total association with retention intention (β = 0.24, *p* = 0.007). Job satisfaction demonstrated a significant positive direct association with retention intention (β = 0.22, *p* = 0.007).

Parenting stress showed no significant direct association with retention intention (β = −0.06, *p* = 0.746), but demonstrated a significant negative indirect association (β = −0.10, *p* = 0.002); its total association was not statistically significant (β = −0.16, *p* = 0.550). Similarly, spousal support showed no significant direct association with retention intention (β = 0.00, *p* = 0.982), but demonstrated a significant positive indirect association (β = 0.22, *p* < 0.001); its total association was not statistically significant (β = 0.22, *p* = 0.064).

Together, work–family balance conflict, resilience, positive affect, job satisfaction, parenting stress, and spousal support accounted for 52% of the variance in retention intention among nurses returning from parental leave (Table 4).

## 4. Discussion

The objective of this study was to test a hypothesized structural model explaining retention intention among clinical nurses returning from parental leave, grounded in the JD–R framework [33]. The respecified model showed improved but marginally acceptable fit relative to the initial hypothesized model and explained 52% of the variance in retention intention. This is comparable to or higher than previous SEM studies reporting 47% of the variance in nurses’ quit intention [34] and 40% in turnover intention among new graduate nurses [35]. The relatively higher explanatory power may reflect the integration of family-related demands and personal and social resources within a single model. However, approximately 48% of the variance remained unexplained, suggesting that organizational factors, such as supervisor support, leadership, staffing adequacy, scheduling flexibility, return-to-work programs, and family-friendly workplace policies, may also be associated with retention intention. Future research should incorporate these organizational resources to develop a more comprehensive model.

Resilience emerged as the strongest positive association with retention intention among nurses returning from parental leave. Within the JD–R framework, resilience can be regarded as a personal resource associated with adaptation to job demands and employee well-being [33]. Notably, resilience showed the largest total association with retention intention in the present study, whereas its direct association was not statistically significant. Resilience demonstrated a statistically significant indirect association with retention intention through the variables (psychological and work-related) specified in the model, including positive affect and job satisfaction.

Returning to clinical practice after parental leave is not simply a resumption of work but a complex process of reintegration and readjustment. During this transition, nurses often experience transition shock, reduced confidence in their clinical competence, emotional exhaustion, and difficulties balancing professional responsibilities with parenting demands [36]. Nurses with higher resilience may be better equipped to adapt to these challenges by maintaining psychological stability, recovering from stressful experiences, and engaging more positively with their work environment. From the perspective of the JD–R framework, these findings are consistent with the view that personal resources are associated with adaptation to job demands and positive psychological states linked to favorable work outcomes.

Work–family balance conflict showed the largest negative total association with retention intention, highlighting its importance as a job demand during the transition back to work after parental leave. This finding is consistent with previous studies showing that work–family balance conflict is an important factor associated with nurses’ turnover intention and retention across different healthcare settings [37].

However, an important finding of this study was that the direct association between work–family balance conflict and retention intention was not statistically significant, whereas its indirect and total associations were significant. This suggests that work–family balance conflict may be associated with retention intention primarily through indirect statistical pathways involving psychological and work-related resources rather than through a direct association. This interpretation is consistent with the JD–R framework, which proposes that excessive job demands influence work-related outcomes by depleting personal resources and impairing motivational processes rather than acting in isolation [38].

Specifically, work–family balance conflict was significantly negatively associated with job satisfaction, whereas its direct association with positive affect was not statistically significant. Nevertheless, its significant indirect association with retention intention suggests a statistical pathway linking work–family balance conflict to retention intention through intervening psychological and work-related processes represented in the model. Previous studies have similarly reported that work–family balance conflict is negatively associated with resilience, psychological well-being, and job satisfaction, all of which are important antecedents of organizational commitment and retention [39,40]. Rather than being directly associated with retention intention, work–family balance conflict was indirectly associated with retention intention through pathways involving psychological and work-related factors.

Characteristics of nursing work, including rotating shifts, overtime, unpredictable workloads, and high emotional demands, may further intensify work–family balance conflict. In Korea, where childcare and domestic responsibilities continue to be disproportionately borne by women, returning nurses may experience substantial psychological and physical burdens while attempting to reconcile rigid work schedules with parenting responsibilities. These findings support previous research emphasizing that organizational demands and inflexible work environments are important contributors to workforce attrition among female healthcare professionals [38].

These findings also suggest that work–family balance conflict should be viewed not merely as an individual coping issue but as a structural and organizational challenge requiring systemic intervention. Organizational efforts to reduce such conflict may play a critical role in improving retention among nurses returning to work. Based on the broader literature, potential strategies may include flexible scheduling, reduced night-shift burden during transition periods, access to childcare support, and family-friendly workplace policies. Fostering a supportive culture in which returning nurses are not stigmatized for taking parental leave may further facilitate reintegration and long-term retention.

An important finding of this study was that spousal support showed a significant indirect, but not direct, association with retention intention. This finding suggests that spousal support showed an indirect statistical association with retention intention involving psychological and work-related variables, although no significant direct association was observed. Previous studies have shown that family support promotes successful work reintegration after parental leave by reducing caregiving burden and enhancing psychological adaptation [41]. Consistent with the JD–R framework, spousal support may be regarded as an external resource associated with resilience and positive psychological and work-related outcomes relevant to retention intention [33].

These findings extend previous research emphasizing the importance of family support during nurses’ return to work after parental leave by demonstrating that its contribution may be indirect rather than direct. From a practical perspective, organizational efforts to support returning nurses should recognize the broader social context in which reintegration occurs. Interventions such as family-inclusive return-to-work education, counseling services, and organizational policies that acknowledge family responsibilities may help reinforce nurses’ psychological resources and facilitate successful workplace reintegration.

Among all variables included in the model, only positive affect and job satisfaction showed significant direct associations with retention intention. This finding suggests that positive emotional experiences and favorable evaluations of one’s work may represent the most proximal factors associated with retention intention among nurses returning from parental leave. Consistent with the broaden-and-build theory, positive affect broadens cognitive flexibility, promotes adaptive coping, and facilitates recovery from stressful experiences, thereby supporting motivation and work engagement [42]. Likewise, job satisfaction has consistently been identified as one of the strongest factors associated with organizational commitment and workforce retention among nurses [43,44].

Importantly, resilience and spousal support showed significant indirect, but not direct, associations with retention intention, indicating indirect statistical associations involving the intervening psychological and work-related variables represented in the model. This pattern is consistent with the JD–R framework, which proposes that personal and contextual resources contribute to favorable work outcomes by fostering motivational and psychological processes [33]. These findings suggest that strengthening nurses’ resilience alone may be insufficient unless it is accompanied by positive emotional experiences and satisfying work environments. Therefore, organizations may consider strategies that not only enhance nurses’ personal resources but also create supportive work environments, including effective leadership, fair workload distribution, flexible work arrangements, and family-friendly workplace policies, as potential approaches to support long-term retention [45,46].

From a nursing management perspective, the present findings should be interpreted cautiously because organizational and managerial factors were not directly examined in this study. Based on the broader literature, structured return-to-work programs, flexible scheduling, supervisor support, and family-friendly workplace policies may be considered as potential strategies to support reintegration and retention [47,48]. These strategies are therefore presented as recommendations informed by the broader literature rather than as interventions whose effectiveness was demonstrated in the present study.

However, these implications should be interpreted in light of the age of the data. The data were collected in 2019, before the COVID-19 pandemic, and changes in nursing practice and work–family conditions since then may limit the direct applicability of the findings to the current nursing workforce. Nevertheless, recent Korean studies of married nurses with preschool-aged children have continued to demonstrate the relevance of work–family conflict, parenting stress, and resilience to retention intention and related work–family experiences [49,50]. Qualitative evidence has also highlighted persistent challenges associated with shift work and childcare responsibilities, as well as the importance of family and organizational support among nurses returning to work after parental leave [51]. Thus, although the magnitude of the relationships observed in 2019 may not directly represent current conditions, the consistency of these issues in subsequent evidence supports the continued conceptual relevance of the factors examined in this study and the JD–R perspective.

Several nonsignificant direct associations also warrant consideration. First, the nonsignificant path from work–family balance conflict to positive affect suggests that nurses may maintain positive emotional states despite role conflict. One possible explanation is that emotional regulation or compartmentalization may help nurses manage competing work and family demands; however, this interpretation remains speculative and was not directly examined in the present study [5,6]. Second, positive affect was not significantly associated with job satisfaction. Possibly, positive affect alone may be insufficient to fully account for job satisfaction and organizational factors may also play an important role during reintegration [6]. Third, the nonsignificant direct association between parenting stress and retention intention may reflect practical or economic considerations not captured in the present model, such as household financial circumstances. However, these factors were not measured in this study, and this explanation should therefore be interpreted cautiously. Fourth, gender-role asymmetry in household responsibilities in Korea may explain the nonsignificant direct association between spousal support and retention intention [46,52]. However, gender-role expectations were not directly assessed in this study, and this interpretation remains tentative. Finally, resilience was not directly associated with retention intention, although a significant indirect association was observed. This finding may suggest that the relationship between resilience and retention intention is more complex than a simple direct association.

This study has some limitations. First, the cross-sectional design precludes causal inference, and longitudinal studies are needed to examine changes in retention intention over time. In addition, participants differed in time since returning to work, which may reflect different stages of psychological and occupational readjustment and may have influenced the observed associations. Second, convenience sampling from 13 general hospitals in four Korean regions may have introduced selection bias and limited generalizability. Institutional and regional differences, including variation in parental leave and return-to-work policies, may have also affected the observed associations. Therefore, the findings should not be generalized to all nurses returning from parental leave in South Korea. Additionally, the findings may not be generalizable to nurses caring for children with chronic health conditions or disabilities, who were excluded from this study. Third, several measurement-related limitations should be considered. The use of self-report questionnaires may have introduced response bias. Although retention intention was modeled as a single latent construct in accordance with the original Nurses Retention Index, organizational retention and professional retention are conceptually distinguishable. Future studies may examine these dimensions separately using measures designed to distinguish intention to remain in the current organization from intention to remain in the nursing profession. In addition, although prior research has provided psychometric support for single-item measures of overall job satisfaction [29], the use of a single item may not fully capture the multidimensional nature of job satisfaction and may introduce measurement error, potentially affecting the estimated structural relationships. Fourth, although the proposed model identified important psychological factors, organizational and managerial variables, such as organizational support and leadership, were not included. Future studies should integrate individual and organizational factors to provide a more comprehensive understanding of retention intention. In addition, the post hoc model modifications may increase the risk of sample-specific model fit, warranting validation of the respecified model in an independent sample. Finally, the data were collected in 2019, before the COVID-19 pandemic. Given subsequent changes in nursing workforce conditions and organizational policies, the magnitude and nature of the observed associations may differ in contemporary nursing environments. Nevertheless, the findings remain theoretically informative within the JD–R framework, although the empirical estimates should be generalized to contemporary nursing contexts with caution. Future studies using more recent and diverse samples, stratifying participants by return-to-work duration and employing validated multi-item measures of job satisfaction, are warranted.

Nevertheless, this study provides important implications by identifying organizational and psychological factors associated with retention intention among nurses returning from parental leave. Resilience showed the strongest positive total association, whereas work–family balance conflict showed the strongest negative total association with retention intention. Positive affect and job satisfaction were the only variables directly associated with retention intention, suggesting that interventions should aim not only to strengthen nurses’ psychological resources but also to foster positive emotional experiences and satisfying work environments. Nursing managers may facilitate successful reintegration through structured return-to-work programs, mentoring systems, flexible work arrangements, adequate staffing, supervisor support, and family-friendly workplace policies. From a research perspective, this study extends the application of the JD–R framework to nurses returning from parental leave by demonstrating both direct and indirect pathways associated with retention intention. Future studies should examine longitudinal changes in adaptation and retention while incorporating organizational and managerial factors, such as organizational support, leadership, and staffing adequacy, to provide a more comprehensive understanding of workforce retention.

## 5. Conclusions

This study tested a hypothesized structural model of retention intention among nurses returning from parental leave based on the Job Demands–Resources (JD–R) framework. The respecified model explained 52% of the variance in retention intention. Resilience showed the strongest positive total association with retention intention, whereas work–family balance conflict showed the strongest negative total association. Positive affect and job satisfaction were the only variables directly associated with retention intention, suggesting that they represent the most proximal factors associated with nurses’ intention to remain after returning from parental leave.

These findings suggest that retention among nurses returning from parental leave should be understood not only as an individual adaptation issue but also as an organizational challenge requiring structural and psychological support. Therefore, organizational efforts to reduce work–family balance conflict and strengthen nurses’ psychological resources may play a critical role in promoting successful workforce reintegration and long-term retention among experienced nurses.

Although healthcare environments have evolved since the data were collected, the JD–R framework remains a useful perspective for understanding factors associated with retention intention among nurses returning from parental leave. Future studies should incorporate organizational and managerial factors, such as organizational support, leadership, and staffing adequacy, while using more recent data to further refine the proposed model in contemporary healthcare settings.

## Figures and Tables

**Table 1:** General Characteristics of the Participants (N = 196).

Characteristics	Categories	N (%) or M ± SD
Age (years)		35.56 ± 4.57
	≤30	25 (12.8)
	31–35	72 (36.7)
	36–39	73 (37.2)
	≥40	26 (13.3)
Education	College	68 (34.7)
	University	105 (53.6)
	≥Graduate school	23 (11.7)
Position	Staff nurse	126 (64.3)
	Charge nurse	50 (25.5)
	≥Unit manager	20 (10.2)
Clinical experience		11.68 ± 4.45
	<5	10 (5.1)
	5–9	58 (29.6)
	10–14	85 (43.4)
	15–19	33 (16.8)
	≥20	10 (5.1)
Number of children	1	116 (59.2)
	2	73 (37.2)
	≥3	7 (3.6)
Parental leave period (months)	<3	23 (11.7)
	3–4	17 (8.7)
	5–6	19 (9.7)
	7–11	56 (28.6)
	≥12	81 (41.3)
Working period after return (years)		1.77 ± 0.76
	<1	84 (42.9)
	1–2	74 (37.8)
	≥3	38 (19.4)

M = Mean; SD = Standard Deviation.

**Table 2:** Descriptive Statistics and Convergent Validity of the Measurement Model (N = 196).

Variables	Descriptive Statistics				Item-Level Statistics			Construct-Level Statistics	
	Mean ± SD	Min–Max	Skewness	Kurtosis	Standardized Factor Loading	S.E.	C.R. (*p*)	CR	AVE
Parenting stress	3.21 ± 0.67	1.47–4.80	−0.08	−0.33				0.89	0.73
Daily stress related to parenting	3.46 ± 0.66	1.67–5.00	−0.29	−0.19	0.78				
Burden and distress related to the parental role	3.10 ± 0.69	1.27–5.00	0.06	−0.31	0.82	0.10	9.83 (<0.001)		
Guilt related to childcare provided by others	2.96 ± 0.83	1.00–5.00	−0.17	−0.52	0.72	0.09	9.51 (<0.001)		
Spousal support	3.66 ± 0.69	1.36–4.93	−0.49	0.25				0.85	0.74
Participation in childcare	3.87 ± 0.84	1.29–5.00	−0.64	−0.08	0.79				
Agreement with spouse on parenting	3.45 ± 0.69	1.43–5.00	−0.14	−0.13	0.79	0.15	8.32 (<0.001)		
Work–family balance conflict	3.34 ± 0.62	1.00–4.70	−0.53	0.10				0.77	0.64
Work-to-family conflict	3.74 ± 0.68	1.00–5.00	−0.72	0.63	0.54				
Family-to-work conflict	2.94 ± 0.79	1.00–4.60	−0.24	−0.64	0.80	0.10	5.80 (<0.001)		
Resilience	3.64 ± 0.50	1.93–4.80	−0.75	1.39				0.97	0.86
Dispositional pattern	3.52 ± 0.64	1.40–5.00	−0.67	0.57	0.83				
Relational pattern	3.71 ± 0.57	1.75–5.00	−0.50	0.89	0.77	0.07	12.21 (<0.001)		
Situational pattern	3.74 ± 0.54	1.80–5.00	−0.89	1.35	0.90	0.06	15.43 (<0.001)		
Philosophical pattern	3.64 ± 0.60	1.50–5.00	−0.31	0.44	0.80	0.07	13.04 (<0.001)		
Professional pattern	3.53 ± 0.54	1.80–5.00	−0.59	0.92	0.81	0.06	13.34 (<0.001)		
Positive affect	2.43 ± 0.73	1.00–4.00	0.05	−0.78				0.89	0.48
Interested	2.32 ± 0.99	1.00–5.00	0.09	−0.83	0.74				
Excited	2.08 ± 0.93	1.00–4.00	0.37	−0.89	0.54	0.09	7.32 (<0.001)		
Strong	2.10 ± 0.97	1.00–4.00	0.25	−1.17	0.70	0.10	9.64 (<0.001)		
Enthusiastic	2.31 ± 1.00	1.00–5.00	0.39	−0.35	0.67	0.10	9.25 (<0.001)		
Determined	2.54 ± 0.96	1.00–5.00	0.09	−0.80	0.54	0.10	7.35 (<0.001)		
Attentive	2.95 ± 0.98	1.00–5.00	−0.11	−0.61	0.53	0.10	7.18 (<0.001)		
Inspired	2.47 ± 1.01	1.00–5.00	0.16	−0.68	0.80	0.10	11.25 (<0.001)		
Active	2.51 ± 1.05	1.00–5.00	0.25	−0.55	0.87	0.10	12.26 (<0.001)		
Proud	2.58 ± 1.05	1.00–5.00	0.08	−0.62	0.79	0.10	11.04 (<0.001)		
Job satisfaction	3.16 ± 0.71	1.00–5.00	−0.08	−0.17					
Retention intention	5.43 ± 1.39	1.17–8.00	−0.47	0.12				0.92	0.66
Item 1	5.84 ± 1.52	1.00–8.00	−0.86	0.78	0.87				
Item 2	5.39 ± 1.77	1.00–8.00	−0.64	−0.14	0.91	0.07	17.68 (<0.001)		
Item 3	5.46 ± 1.62	1.00–8.00	−0.36	−0.58	0.78	0.07	13.94 (<0.001)		
Item 4	5.26 ± 1.75	1.00–8.00	−0.44	−0.34	0.84	0.07	15.40 (<0.001)		
Item 5	5.86 ± 1.42	1.00–8.00	−1.01	1.42	0.85	0.06	15.64 (<0.001)		
Item 6	4.77 ± 1.87	1.00–8.00	−0.11	−0.73	0.56	0.09	9.02 (<0.001)		

S.E. = Standard Error; C.R. = Critical Ratio; CR = Construct Reliability; AVE = Average Variance Extracted.

**Table 3:** Goodness of Fit Indices for the Hypothesized and Respecified Models (N = 196).

Goodness	χ^2^ (*p*)	χ^2^/df	RMR	SRMR	RMSEA	GFI	NFI	TLI	CFI
Hypothesized Model	714.327 (<0.001)	2.132	0.075	0.069	0.076	0.779	0.809	0.872	0.887
Respecified model	667.252 (<0.001)	2.004	0.070	0.067	0.072	0.798	0.821	0.887	0.900

CFI = Comparative fit index; NFI = Normed fit index; TLI = Tucker–Lewis index; GFI = Goodness-of-fit index; RMSEA = Root mean square error of approximation; RMR = Root mean square residual; SRMR = Standardized root mean square residual.

**Table 4:** Hypothesis Testing and Standardized Effects of the Respecified Model (N = 196).

Endogenous Variables Exogenous Variables	Hypothesis	Decision	Direct Effect (*p*) [95% BC CI]	Indirect Effect (*p*) [95% BC CI]	Total Effect (*p*) [95% BC CI]	Squared Multiple Correlations
Resilience						0.48
Parenting stress	H1	Supported	−0.25 (0.003) [−0.42, −0.09]		−0.25 (0.003) [−0.42, −0.09]	
Spousal support	H2	Supported	0.55 (<0.001) [0.34, 0.70]		0.55 (<0.001) [0.34, 0.70]	
Positive affect						0.34
Parenting stress				−0.13 (0.002) [−0.27, −0.05]	−0.13 (0.002) [−0.27, −0.05]	
Spousal support				0.29 (<0.001) [0.19, 0.41]	0.29 (<0.001) [0.19, 0.41]	
Work–family balance conflict	H3	Not supported	−0.10 (0.483) [−0.32, 0.13]		−0.10 (0.483) [−0.32, 0.13]	
Resilience	H4	Supported	0.53 (<0.001) [0.37, 0.67]		0.53 (<0.001) [0.37, 0.67]	
Job satisfaction						0.26
Parenting stress				−0.08 (0.002) [−0.19, −0.03]	−0.08 (0.002) [−0.19, −0.03]	
Spousal support				0.18 (0.001) [0.09, 0.28]	0.18 (0.001) [0.09, 0.28]	
Work–family balance conflict	H5	Supported	−0.25 (0.019) [−0.46, −0.03]	−0.01 (0.272) [−0.08, 0.01]	−0.27 (0.025) [−0.46, −0.03]	
Resilience	H6	Supported	0.24 (0.007) [0.07, 0.40]	0.08 (0.074) [−0.01, 0.20]	0.32 (0.001) [0.16, 0.48]	
Positive affect	H7	Not supported	0.15 (0.086) [−0.02, 0.32]		0.15 (0.086) [−0.02, 0.32]	
Retention intention						0.52
Parenting stress	H8	Not supported	−0.06 (0.746) [−0.36, 1.06]	−0.10 (0.002) [−0.23, −0.03]	−0.16 (0.550) [−0.51, 1.01]	
Spousal support	H9	Not supported	0.00 (0.982) [−0.25, 0.26]	0.22 (<0.001) [0.11, 0.37]	0.22 (0.064) [−0.02, 0.43]	
Work–family balance conflict	H10	Not supported	−0.27 (0.091) [−1.53, 0.03]	−0.08 (0.031) [−0.18, −0.01]	−0.34 (0.033) [−1.64, −0.01]	
Resilience	H11	Not supported	0.22 (0.087) [−0.04, 0.47]	0.18 (<0.001) [0.07, 0.31]	0.40 (0.001) [0.16, 0.61]	
Positive affect	H12	Supported	0.21 (0.014) [0.05, 0.37]	0.03 (0.052) [0.00, 0.09]	0.24 (0.007) [0.08, 0.41]	
Job satisfaction	H13	Supported	0.22 (0.007) [0.07, 0.35]		0.22 (0.007) [0.07, 0.35]	

BC CI = Bias-Corrected Confidence Interval; H = Hypothesis.

## Data Availability

The data presented in this study are available on request from the corresponding author. The data are not publicly available due to privacy concerns.

## Abbreviations

The following abbreviations are used in this manuscript:

JD–R	Job Demands–Resources
